# Digital, remotely delivered management interventions for adult asthma: a systematic review and meta-analysis

**DOI:** 10.3389/fpubh.2026.1776893

**Published:** 2026-03-31

**Authors:** Shan Tian, Xiaonan Huang, Jian Li, Caitao Chen, Hong Zhang

**Affiliations:** 1 College of Acupuncture and Tuina, Chengdu University of Traditional Chinese Medicine, Chengdu, China; 2 Department of Rehabilitation Medicine, Changhai Hospital, Naval Medical University, Shanghai, China; 3 School of Rehabilitation Medicine, Shanghai University of Traditional Chinese Medicine, Shanghai, China; 4 Department of Rehabilitation Medicine, Shanghai Fourth People's Hospital Affiliated to Tongji University, Shanghai, China; 5 College of Sport Rehabilitation, Shanghai University of Sport, Shanghai, China

**Keywords:** asthma, mini-asthmaquality of life questionnaire, mobile health, remote digital management, telemedicine

## Abstract

**Background:**

Remote, digitally delivered asthma management is increasingly used in routine care, but its long-term impact on core clinical outcomes in adults remains uncertain.

**Methods:**

We conducted a systematic review and meta-analysis of randomized controlled trials evaluating interactive remote digital management (smartphone applications or web-based platforms) vs. usual care in adults with physician-diagnosed asthma and ≥6 months follow-up. Primary outcome was Mini Asthma Quality of Life Questionnaire (mini-AQLQ) at 6 and 12 months. Secondary outcomes included Asthma Control Questionnaire (ACQ) at 12 months, FEV_1_% predicted at 12 months. Random-effects models generated pooled mean differences (MD) or risk ratios.

**Results:**

Ten trials were included. Remote digital interventions improved mini-AQLQ at 6 months (MD 0.47, 95% CI 0.02 to 0.92) and 12 months (MD 0.35, 95% CI: 0.21 to 0.49) and ACQ at 12 months (MD −0.37, 95% CI: −0.62 to 0.13). FEV_1_% predicted increased at 12 months (MD 4.49%, 95% CI: 1.11 to 7.87). Subgroup analyses suggested more consistent benefits for web-based programs than for app-based interventions. GRADE rated evidence as high for FEV_1_% predicted, moderate for 12-month mini-AQLQ, and very low for ACQ and 6-month mini-AQLQ due to risk of bias and inconsistency.

**Conclusions:**

Interactive remote digital management statistically significant but modest improvements in quality of life and symptom control, which did not reach the established MCID of 0.5. These findings support structured, clinician-supported digital interventions as a useful, though limited, adjunct to standard pharmacologic management for adult asthma.

**Systematic Review Registration:**

https://www.crd.york.ac.uk/PROSPERO/view/CRD420251133851, identifier: CRD420251133851.

## Introduction

Asthma remains one of the most prevalent chronic airway diseases worldwide, imposing a sustained medical and socioeconomic burden on patients and healthcare systems. Current estimates suggest that approximately 260 million people are living with asthma, with 450,000–460,000 asthma-related deaths each year, the majority occurring in low- and middle-income countries (1). The economic impact is similarly substantial: direct healthcare costs over the next two decades are projected to reach several hundred billion dollars (2); When assessed using a comprehensive economic evaluation, the overall economic burden is expected to escalate further, underscoring the urgent need for effective, accessible, and sustainable long-term management strategies (3).

Against this backdrop, remote digital management approaches—such as smartphone applications and web-based platforms—often integrated with digital inhalers or other electronic monitoring devices, provide a novel closed-loop pathway for asthma care, an integrated system where patient monitoring data directly informs timely clinical feedback. In real-world settings, these technologies enable continuous collection of multidimensional data, including symptom trajectories, medication use, and lifestyle behaviors (4); By leveraging AI-driven individualized feedback together with remote follow-up by healthcare professionals, they extend the “assessment–intervention–reassessment” cycle from hospitals into patients' homes and communities, thereby enhancing treatment coverage, adherence, and continuity of care (5). As of 2024, approximately 68% of the global population has internet access, and the rapid expansion of mobile networks and smart devices offers critical infrastructure to support large-scale deployment of remote digital health solutions, helping to narrow urban–rural disparities in access to care (6); In parallel, advances in the mobile ecosystem and AI technologies have enabled automated alerts, risk stratification, and behavior-change interventions, further reinforcing the feasibility and scalability of such strategies (7).

Emerging evidence suggests that remote digital management may confer benefits across multiple clinically relevant domains. Digital inhalers have been shown to significantly improve adherence to maintenance therapy in the short term while maintaining or enhancing asthma control during follow-up (8); Similarly, self-management interventions delivered via smartphone applications or web-based platforms have been associated with improvements in symptom control, lung function, and medication adherence (9); Among individuals with difficult-to-control asthma, remote management strategies incorporating digital inhalers may also be cost-effective for long-term care (10). Nevertheless, heterogeneity in intervention components, follow-up durations, target populations, and outcome measures limits the certainty and generalizability of current evidence.

Existing studies can broadly be categorized into two streams. The first focuses predominantly on remote self-management education and often lacks fully integrated, closed-loop remote management involving bidirectional interaction with healthcare professionals and interoperable digital devices (9); The second is derived largely from experience with remote monitoring in chronic obstructive pulmonary disease (COPD), which cannot be directly extrapolated to adult asthma due to important pathophysiological and clinical differences (11, 12); Furthermore, previous reviews have frequently prioritized adherence as the principal outcome, while quantitative evaluations of key asthma-specific endpoints, such as symptom control and lung function, remain insufficient (13). Therefore, the present study concentrates on remote digital management interventions for adult asthma and systematically evaluates their impact on core clinical outcomes, including the Asthma Control Questionnaire (ACQ), the Asthma Quality of Life Questionnaire (AQLQ), and forced expiratory volume in one second (FEV1), with the aim of generating more robust and practice-oriented evidence to support long-term standardized asthma management in outpatient and home-based settings. Beyond clinical efficacy, this review also characterizes the included digital interventions in terms of their development source, engagement requirements, and usability, to identify specific implementation features that may influence long-term adherence and outcomes.

## Methods

### Search strategy

We systematically searched PubMed, the Cochrane Library, Web of Science, Scopus, and Embase on September 12, 2025, without restrictions on publication date. This review was prospectively registered in PROSPERO on August 25, 2025 (Registration number: CRD420251133851). The detailed search strategies for each database are presented in Supplementary Table 1.

### Eligibility criteria

Studies were selected according to the following PICOS framework:

Patients: Adults (≥18 years) with physician-diagnosed asthma. No restrictions were placed on sex, race/ethnicity, or asthma severity; Interventions: Remote, digital software or web-based platforms—optionally integrated with digital or connected inhalers—used as core components to remotely record symptoms, deliver interventions, and conduct follow-up for asthma management. We excluded interventions that only collected data or provided educational content via applications or websites without interactive engagement, as well as those delivered exclusively through traditional modalities such as SMS or telephone; Comparison: Usual care or alternative interventions that fulfilled the above criteria; Outcomes: Studies were required to report at least one asthma-specific questionnaire (e.g., AQLQ or ACQ) and pulmonary function outcomes such as FEV_1_% predicted, with a minimum follow-up duration of 6 months and outcome data reported at 6 or 12 months; Study design: Randomized controlled trials (RCTs). Retrospective studies, cross-sectional studies, cohort studies, and systematic reviews were excluded.

### Data extraction and assessment of risk of bias

Two reviewers (HXN and TS) independently screened titles and abstracts in EndNote. Any discrepancies were resolved through discussion with a third reviewer (ZH). Data were extracted using a standardized Excel spreadsheet and included baseline characteristics, inclusion and exclusion criteria, detailed descriptions of the interventions and comparators, and outcome measures at 6 and 12 months for each study arm. Risk of bias was assessed at the study level using the Cochrane Risk of Bias 2 (ROB 2) tool (14). The certainty of evidence for each outcome was evaluated according to the Grading of Recommendations, Assessment, Development, and Evaluation (GRADE) approach and summarized using GRADEpro GDT (http://www.guidelinedevelopment.org) (15). When studies reported medians with interquartile ranges, these were converted to means and standard deviations using established methods (16). AQLQ scores were linearly rescaled to the mini-AQLQ metric where necessary to ensure consistency across studies.

### Outcomes

The primary outcome was the mini-AQLQ score at 6 and 12 months. Secondary outcomes included the ACQ score at 12 months, FEV_1_% predicted at 12 months, and the attrition rate.

### Statistical analysis

We reported continuous outcomes as mean differences (MD) with 95% confidence intervals (CI) and dichotomous outcomes as risk ratios (RR) with 95% CIs. Statistical heterogeneity was assessed using the I^2^ statistic. All pooled analyses were conducted using random-effects models in R (version 4.3.3) with the meta package. Forest plots were used to present pooled effect estimates, and funnel plots were used to explore potential publication bias (17). Subgroup analyses were performed according to delivery modality (smartphone applications vs. web-based platforms) to examine potential differences in treatment efficacy and adherence, using the same statistical methods described above. Sensitivity analyses were not conducted due to the limited number of included studies. Finally, a leave-one-out sensitivity analysis was performed for outcomes with significant heterogeneity to evaluate the robustness of the results.

## Results

### Study selection and population characteristics

**Figure 1:**
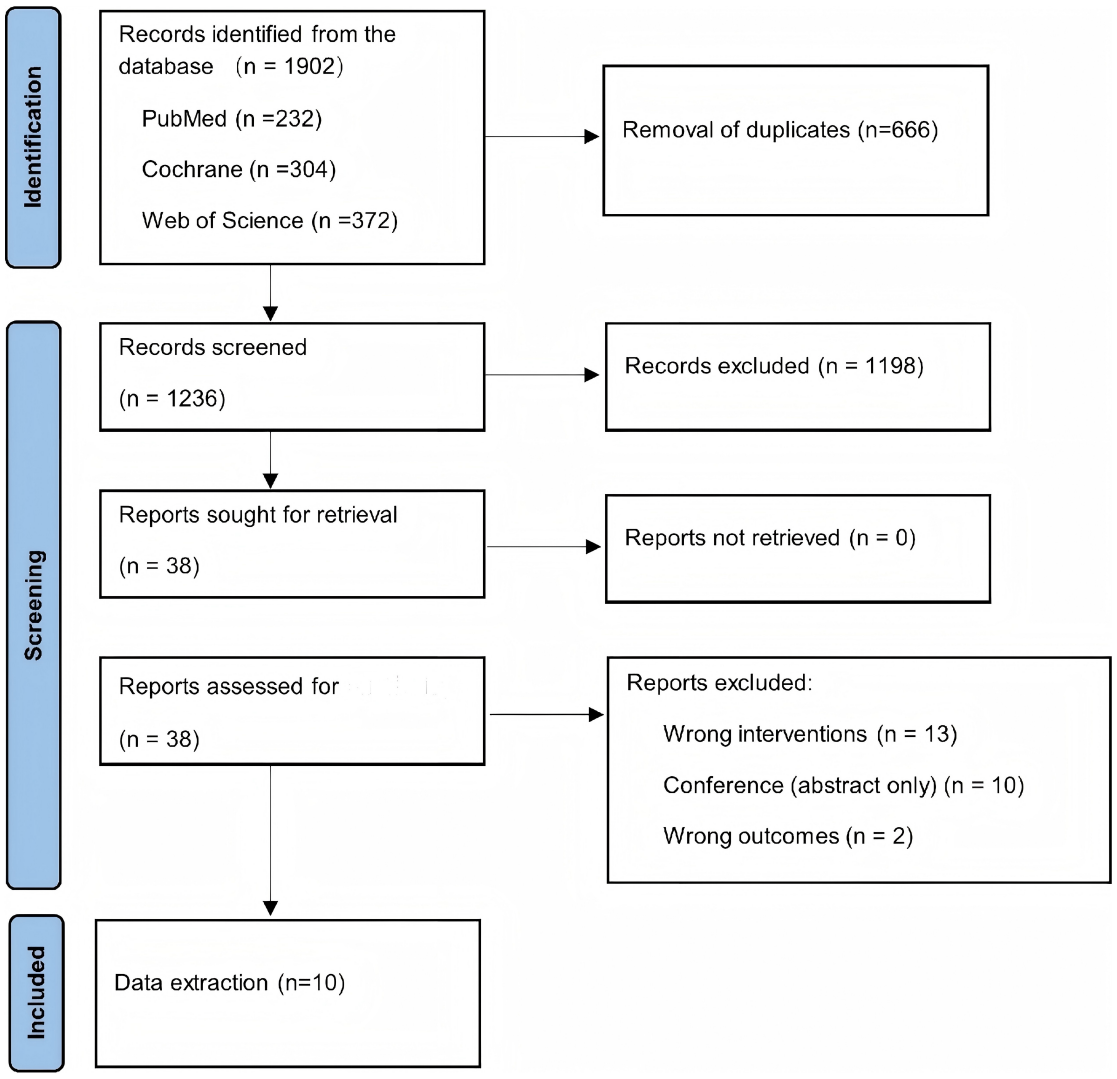
PRISMA flow diagram for the literature search and study selection.

A total of 1,902 records were identified through database searching, of which 666 were removed as duplicates. Following title and abstract screening, 38 articles were selected for full-text assessment, and 10 randomized controlled trials were ultimately included (10, 18–26) (Figure 1). supplementary Table 2 summarizes the studies excluded from the original search and the reasons for their exclusion. Among these, 6 studies evaluated smartphone application-based interventions and 4 evaluated web-based interventions, comprising a total of 1,537 adults with asthma. Two trials were multicenter and eight were single-center. Only 1 trial implemented a combined intervention involving both a smartphone application and a digital inhaler. The key characteristics of the included studies are summarized in Table 1.

**Table 1:** Characteristics of included studies and digital interventions.

Study ID	Study population	Study population	Intervention details	Outcomes	Follow-up period
Xu 2025	Adults with moderate–severe asthma, outpatient setting	Mobile APP mini program follow-up vs. routine face-to-face care	Software: WeChat Mini Program “Asthma Self-management.” Core modules: (1) Education library (articles + inhaler technique videos); (2) Symptom/medication diary incl. PEF; (3) ACT e-questionnaire; (4) Online consultation portal. Automation & prompts: Daily auto-reminders for inhalation & diary; weekly prompt for ACT. Data I/O: Patients submit text/image/video. Human oversight: Pharmacist monitors entries and provides phone/WeChat feedback during the initial 3-month phase. Control arm: Standard face-to-face education + paper diary; monthly 20–30 min pharmacy visits in first 3 months	Primary: ACT. Secondary: FEV_1_% predicted, ASMQ, AQLQ, ED visits/hospitalizations.	6 months
Susanne 2025	Adults with doctor-diagnosed, suboptimally controlled asthma (ACQ-5 ≥ 0.75)	Mobile APP + remote digital inhaler program vs. passive electronic monitoring	Hardware: EMD Device attached to SYMBICORT Turbuhaler; Bluetooth to phone. smartphone app showing inhaler-use visualizations; configurable reminders, missed-dose and overuse alerts; motivational “nudge” voice messages; optional symptom/trigger tracking. Web portal allowing clinicians to view actuation data when the general practice participated. Control arm: EMD connected to Hailie Lite app with no actuation visibility to patients; GPs followed usual care.	Primary: Medication adherence. Secondary: Shift to adherent; zero-adherent days; underuse days; ACQ-5; Mini-AQLQ; System usability scale; cost-effectiveness.	12 months
Robert 2025	Adults with EHR-identified asthma in 7 primary-care clinics at Brigham & Women's Hospital (Mass General Brigham), US	EHR-integrated between-visit symptom monitoring via smartphone app vs. usual care (general asthma guidance).	Software: Smartphone app. Core tasks: weekly 5-item asthma symptom questionnaire; optional notes, peak-flow and trigger tracking; symptom graphs; education; visit reminders. Clinical integration: patient-entered data written to the EHR; PCP-facing EHR dashboard shows patient trends with inbox reminders prior to scheduled visits; nurse-facing practice model routes EHR inbox messages for callback requests to a nurse/nurse pool with nurse-driven triage protocol. Usage: patients asked to download/use app on their own phones; entirely remote except some in-clinic recruitment. Control arm: email with general asthma advice; otherwise usual care.	Primary: Mini-AQLQ. Secondary: Non-routine asthma-related health-care utilization.	12 months
Mehrdad 2024	Adults with intermittent–severe persistent asthma per GINA	Smartphone self-management app + usual care vs. usual care alone	Software: RespRight Android app. Core functions: PEFR and symptom logging; guideline-based education (inhaler technique, trigger control); medication reminder alerts; in-app feedback messages on entered PEFR trends; patient-to-physician communication channel. Data I/O & devices: Manual entry of peak-flow values. Both groups received baseline face-to-face education and individualized action plans; controls recorded PEFR manually without app support.	Primary: ACT. Secondary: AQLQ	6 months
Beerthuizen 2020	Adults with severe asthma completing a 12-week high-altitude pulmonary rehabilitation program at the Dutch Asthma Center Davos	Mobile app-based self-management support + usual care vs. usual care alone	Software: PatientCoach web/app platform. Modules: written action plan (tailored alarm signals & when-to-seek-care directives); daily ACQ+ FEV_1_ entry; personalized control question; education library; calendar. Devices & I/O: handheld spirometer PIKO-1 given to intervention patients to measure FEV_1_ at home; Fitbit Ultra actometer for daily steps with personal step-goals; patient self-entry of FEV_1_/ACQ; no provider messaging within the app. Tailoring & autonomy: patient-specific ACQ/FEV_1_ cutoffs and goals set with rehab physician. Control arm: usual post-discharge care by own physicians; no access to PatientCoach.	Primary: AQLQ. Secondary: ACQ; eHealth engagement; Health Education Impact Questionnaire domains.	12 months
Ben 2019	Adults (18–75 y) with physician-confirmed asthma from a Slovenian tertiary clinic; ≥ 1 exacerbation in prior year or persistent symptoms	Telemonitoring with automated triage & nurse callbacks vs. usual care.	Software: Web-based application + SMS. Patient data entry: ACT as two-digit number; PEF as three-digit value. Automated reminders: SMS prompts weekly and ACT monthly; extra reminder if no data within 48 h. Thresholds & alerts: Critical values set at PEF < 80% of personal best or ACT < 20 → automatic alert to study nurse. Human oversight & escalation: Study nurse checks the web app 3 × /workday, calls patient to verify values/reasons, and involves pulmonologist by phone or books a visit as needed. Control arm: No SMS/web access; both groups continued routine pulmonology care with 3–6-month clinic visits.	Primary: Change in ACT. Secondary: Exacerbations per patient/year; lung function; patient-reported program effects.	12 months
Tiva 2019	Adults with physician-diagnosed asthma; impaired asthma-specific QoL (Mini-AQLQ < 5.5)	Web-based self-management program + usual care vs. usual care + Asthma UK booklet	Software: Web intervention built with LifeGuide. Content modules: tailored pharmacological and non-pharmacological support. Automation & prompts: tailored advice after onboarding; automated reminders when users had not accessed the site for several weeks or when new content became available. Engagement support: if no log-in within 1 month, send one email + one phone call offering technical help. Control arm: standardized Asthma UK booklet.	Mini-AQLQ, ACQ; lung function (FEV_1_, FEV_1_/FVC); healthcare utilization from records.	12 months
Sara 2016	Adults 18–69 with physician-diagnosed asthma from 2 tertiary clinics (Montreal, Canada)	Web-based self-management patient portal + nurse case-management vs. usual care.	Software: My Asthma Portal website linked to MOXXI EHR/administrative data. Core functions: weekly log-in request; symptom & behavior monitoring, learning center, individualized action plan; color-coded feedback based on Canadian guidelines. Automation & alerts: email alerts for suboptimal control, action-plan updates, or no log-in ≥ 7 days. Clinical oversight & escalation: nurse reviews flagged cases, contacts patient within 24 h via portal/phone, documents advice. Data I/O: patient-entered monitoring + administrative pharmacy/ED data fed from MOXXI to drive feedback. Control: usual pulmonology care and asthma-nurse education/calls as needed; no portal access.	Primary: Asthma control; mini-AQLQ. Secondary: ACT, PHQ-9, Chronic Disease Self-Efficacy, Beliefs About Medicines Questionnaire.	9 months
Johanna 2013	Adults (18–50 y) with physician-diagnosed asthma, ICS ≥ 3 months in prior year.	Internet-based self-management + usual care vs. usual care alone.	Software: Web portal + optional SMS. Monitoring cadence: weekly ACQ and FEV1 entries; daily day/night symptom scores during setup; reminder messages by email. Devices/Data I/O: handheld electronic spirometer for home FEV1; patient self-entry via website. Human oversight: asynchronous e-messaging/phone/Web communication with a respiratory nurse; Education: online pages/newsfeed + group education session. Control: routine care.	AQLQ, ACQ, FEV1, daily ICS dose	30 months
Victor 2009	Adults aged 18–50	Internet-based self-management + education vs. usual physician-provided care	Software: web portal. Devices/Data I/O: electronic spirometer for FEV1; patient self-entry via web/SMS. Monitoring cadence: weekly ACQ required; optional daily symptoms + morning pre-medication FEV1. Decision support: instant algorithmic feedback with step-up/down advice based on ACQ thresholds; 4-week evaluation period after step-up; “optimal control” period logic for step-down. Education & HCP interaction: online education pages plus two 45–60 min group sessions within 6 weeks; remote web messaging with specialized asthma nurse. Control arm: usual care; web diary access only during assessment windows.	Primary: AQLQ. Secondary: ACQ, FEV1, exacerbations.	12 months

ACT, Asthma Control Test; AQLQ, Asthma Quality of Life Questionnaire; ASMQ, Asthma Self-Management Questionnaire; ACQ, Asthma Control Questionnaire; FEV1, forced expiratory volume in 1 second; ED, emergency department.

In the remote-intervention group, 690 of 774 participants completed the trial (89.14%), compared with 702 of 763 participants in the usual-care control group (92.00%). The corresponding mean ages were 45.43 and 45.83 years. Among studies reporting sex distribution, only 1 had a male proportion exceeding 50%; all others were predominantly female, with 5 studies including more than 70% female participants (supplementary Table 3).

### Risk of bias within studies and quality assessment

The risk-of-bias assessment of the randomized controlled trials indicated that 6 studies had an overall high risk of bias, primarily due to limitations in participant blinding that could affect outcome assessment, as well as missing data and reporting issues among participants who did not adhere to study procedures. Eight studies were judged to be at high risk in the domain “bias in measurement of the outcome,” which contributed substantially to the high overall risk of bias (Supplementary Figures 1, 2).

### Mini-AQLQ, ACQ and FEV_1_% predicted changes

**Figure 2:**
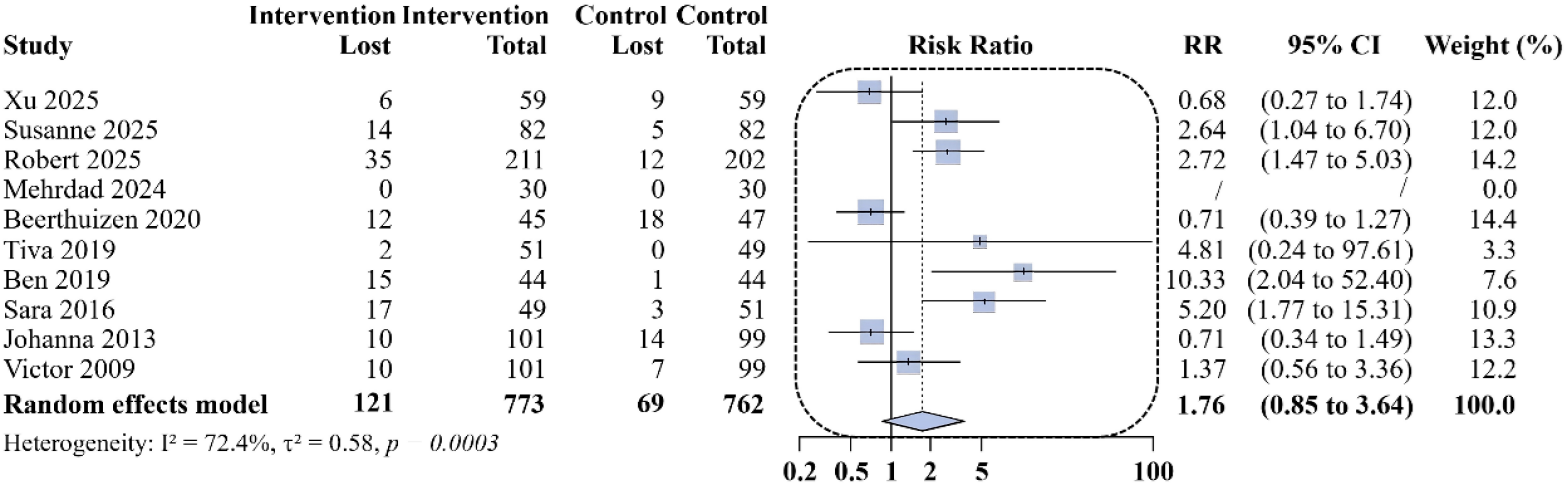
Differences in changes in mini-AQLQ scores at 6 and 12 months of follow-up between the remote intervention group and the standard control group.

Among the 10 included studies, five (10, 18, 20, 22, 24) reported mini-AQLQ scores at 6 months, The pooled analysis demonstrated a mean difference of 0.47 points in favor of remote interventions compared with usual care (95% CI: 0.02 to 0.92; *I*^2^ = 70.6%; Figure 2). At 12 months, 6 studies (10, 19, 21, 23, 25, 26) reported mini-AQLQ scores, with a mean difference of 0.35 points (95%CI: 0.21 to 0.49; *I*^2^ = 43.3%). Overall, remote digital interventions were associated with improvements in quality of life at both 6 and 12 months.

**Figure 3:**
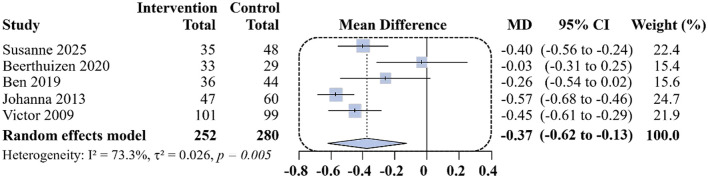
Differences in ACQ score changes between the remote intervention group and the standard control group at 12 months follow-up.

Among the 10 studies included in this meta-analysis, 5 studies (10, 21, 23, 25, 26) reported ACQ scores at 12 months, The pooled mean difference between the remote-intervention and usual-care groups was −0.37 points (95% CI: −0.62 to −0.13; *I*^2^ = 73.3%; Figure 3). As lower ACQ scores indicate better asthma control, this finding suggests a statistically significant benefit of remote interventions, although the presence of substantial heterogeneity warrants cautious interpretation.

**Figure 4:**
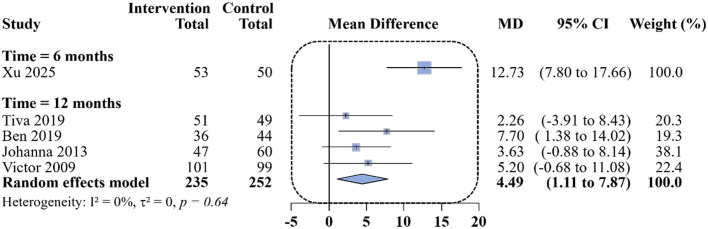
Differences in changes in FEV_1_% Predicted at 6 and 12 months of follow-up between the remote intervention group and the standard control group.

Regarding lung function, 1 study reported FEV_1_% predicted at 6 months (18), demonstrating a MD of 12.73% in favor of remote interventions (95% CI: 7.8% to 17.66%). At 12 months, 4 studies (22, 23, 25, 26) reported FEV_1_% Predicted, with a pooled MD of 4.49% (95% CI: 1.11% to 7.87%; *I*^2^ = 0%; Figure 4). Although FEV_1_ measurements can be influenced by test variability, the high certainty of evidence according to the GRADE assessment and the lack of statistical heterogeneity (*I*^2^ = 0%) across the included trials suggest that the observed 4.49% increase reflects a consistent physiological improvement rather than random measurement error. These findings indicate a small-to-moderate but statistically significant improvement in lung function at 1 year associated with remote digital interventions. Notably, although the 4.49% increase in FEV_1_% predicted was statistically significant, it remains below the 15% threshold recommended by ATS/ERS for a clinically significant change in individual lung function (27). This indicates that while remote digital management provides a consistent physiological benefit at the group level, the magnitude of effect is modest and may only reach clinical importance in a specific subgroup of patients.

### Comparison of study loss to follow-up rates

**Figure 5:**
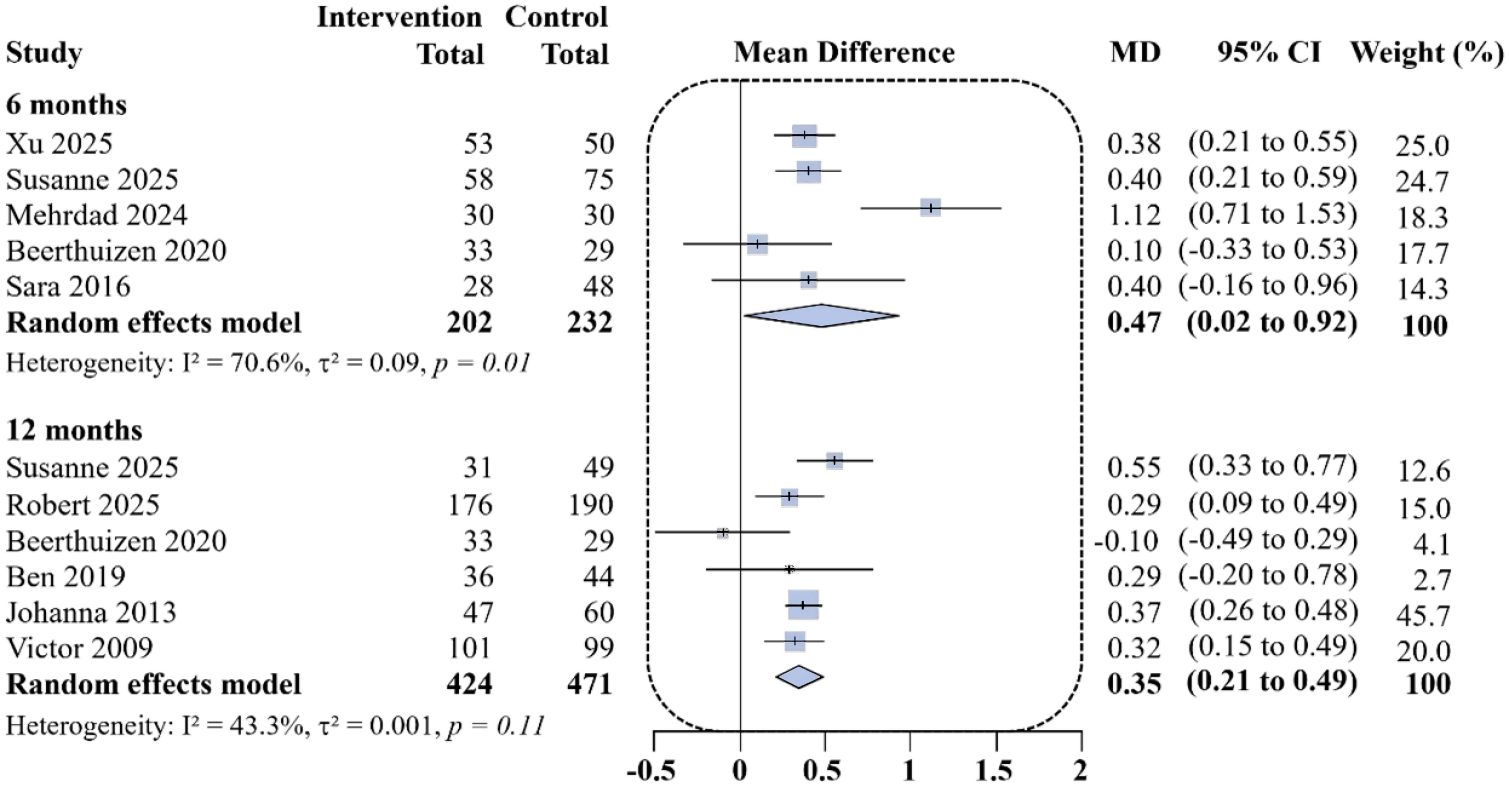
Difference in loss to follow-up rate between remote intervention group and conventional control group.

Across the 10 studies, attrition was assessed at each trial's final follow-up time point. The overall attrition rate was 15.65% in the remote-intervention group and 9.06% in the usual-care group. The pooled risk ratio (RR) comparing remote interventions with usual care was 1.76 (95% CI: 0.85 to 3.64; *I*^2^ = 72.4%; Figure 5). Trial completion was numerically higher in the usual-care group than in the remote-intervention group.

### Subgroup analysis results

Because fewer than 2 eligible studies were available for web-based interventions reporting mini-AQLQ scores at 6 months and for app-based interventions reporting FEV_1_% predicted at 12 months, subgroup analyses were not performed for these outcomes. For mini-AQLQ at 12 months, subgroup analysis showed that the difference between the app-based remote-intervention group and the usual-care group was 0.28 (95% CI: −0.5 to 1.06), with no statistically significant effect (Supplementary Figure 3). In contrast, the web-based remote-intervention group demonstrated a significant improvement compared with usual care, with a mean difference of 0.35 (95% CI: 0.28 to 0.43; Supplementary Figure 4); For ACQ scores at 12 months, the app-based subgroup showed a mean difference of 0.23 (95% CI: −2.57 to 2.1) vs. usual care, indicating no significant difference (Supplementary Figure 5). whereas the web-based subgroup showed a significant benefit of remote interventions, with a mean difference of −0.47 points (95% CI: −0.82 to −0.11; Supplementary Figure 6); Finally, subgroup analyses of attrition by delivery modality revealed no significant differences between intervention and control groups in either subgroup (Supplementary Figures 7S, 8S).

### GRADE assessment of evidence

Based on the GRADE assessment, smartphone app and web-based interventions for adults with asthma demonstrate clinically meaningful advantages over usual care in lung function and long-term quality of life. The certainty of evidence for FEV_1_% predicted was high (5 RCTs; intervention *n* = 235, control *n* = 252; MD = 4.49, 95% CI: 1.11 to 7.87), indicating a small-to-moderate improvement in lung function. For mini-AQLQ at 12 months, the certainty of evidence was moderate (6 RCTs; *n* = 424 vs. 471; MD = 0.35, 95% CI: 0.21 to 0.49). In contrast, the evidence for ACQ at 12 months (5 RCTs; *n* = 252 vs. 280; MD = −0.37, 95% CI: −0.67 to −0.13) and for mini-AQLQ at 6 months (5 RCTs; *n* = 202 vs. 232; MD = 0.47, 95% CI: 0.02 to 0.92) was downgraded to very low, primarily due to risk of bias, inconsistency, and imprecision. Overall, remote digital interventions were associated with consistent, robust improvements in lung function and in quality of life at one year, whereas short-term effects based on patient-reported outcomes remained unstable and require confirmation in future high-quality, methodologically rigorous randomized controlled trials. A summary of the GRADE findings is provided in Supplementary Table 4.

### Publication bias

We assessed publication bias for mini-AQLQ at 6 and 12 months, ACQ at 12 months, and FEV_1_% predicted at 12 months (Supplementary Figures 9–11). For mini-AQLQ, Egger's regression test showed a z-value of 0.228 (*p* = 0.82) at 6 months and −1.19 (*p* = 0.23) at 12 months; For ACQ scores at 12 months, Egger's regression intercept yielded a z-value of 3.36 (*p* = 0.003); For FEV_1_% predicted at 12 months, the z-value was 0.525 (*p* = 0.6). Taken together, these findings suggest no significant publication bias for any outcome except ACQ at 12 months.

Based on the consistency results, sensitivity analyses were performed for the mini-AQLQ scores at 6 and 12 months, as well as the ACQ scores at 12 months. Regarding the mini-AQLQ at 6 months, substantial heterogeneity was initially observed (*I*^2^ = 70.6%). Sensitivity analysis identified the study by Mehrdad et al. (20) as the primary source of this heterogeneity. Upon its exclusion, *I*^2^ decreased to 0% while the pooled mean difference (MD) remained statistically significant and precise (MD = 0.37; 95% CI: 0.22 to 0.51), indicating high consistency among the remaining four studies (Supplementary Figure 12). For the mini-AQLQ at 12 months, the leave-one-out sensitivity analysis confirmed the robustness of the primary findings. Across all iterations, the pooled MD remained stable, ranging from 0.31 to 0.37, maintaining statistical significance throughout. The study by Beerthuizen et al. (21) was identified as the sole source of heterogeneity (21); its removal reduced *I*^2^ from 43.3% to 0.0% and slightly increased the pooled effect estimate (MD = 0.37; 95% CI: 0.27 to 0.47; Supplementary Figure 13). Finally, for the ACQ scores at 12 months, the study by Beerthuizen et al. (21) was found to be the largest contributor to the observed heterogeneity (21). Excluding this study reduced *I*^2^ from 73.3% to 49.9% and strengthened the pooled treatment effect (MD = −0.45; 95% CI: −0.64 to −0.27; Supplementary Figure 14).

## Discussion

In the 10 included RCTs (*n* = 1,537), remote digital management interventions produced small-to-moderate improvements in asthma control and lung function compared with usual care. Mini-AQLQ scores improved at both 6 and 12 months, and ACQ scores decreased, indicating better asthma control. Lung function also improved, as reflected by an increase in FEV_1_% predicted at 12 months. Overall, across a 6- to 12-month observation period, remote digital interventions demonstrated statistically significant and directionally consistent benefits for symptoms, quality of life, and lung function, providing supportive evidence for their use in long-term outpatient and home-based asthma management. While the statistical significance of these improvements is robust, the clinical magnitude warrants a cautious interpretation, as the pooled MDs for mini-AQLQ and ACQ-5 were below the 0.5 MCID. This may be due to the inclusion of patients with varying baseline control levels. However, the concurrent improvement in objective FEV_1_% predicted (MD 4.49%) suggests that these digital tools provide physiological benefits that complement patient-reported outcomes.

Based on the subgroup analyses, at 12 months, web-based interventions were associated with greater improvements in mini-AQLQ and ACQ scores, whereas app-based interventions did not reach statistical significance. However, all four web-based studies were published in 2019 or earlier, while all app-based studies were published after 2019. This pattern may reflect that earlier web-based interventions typically adopted “multi-component, high-intensity programs,” in addition to weekly or bi-weekly online monitoring, they frequently incorporated guideline-based medication advice, structured educational modules, and case management (25). Moreover, web-based platforms are inherently well suited to regular tasks such as behavioral plan adjustment and treatment optimization, whereas smartphone applications despite enabling higher-frequency interaction tend to be more fragmented and better aligned with day-to-day self-management. In the absence of clinician prompts and staff support, engagement and adherence with app-based interventions are more likely to decline over time (28, 29). Differences in baseline severity may also contribute to the observed divergence: 2 of the 4 web-based studies predominantly enrolled participants with mild-to-moderate symptoms (25, 26), whereas 2 of the 6 app-based studies enrolled patients with moderate-to-severe asthma (10, 18), and one specifically targeted individuals with severe disease (21). Thus, the comparatively smaller effects observed in app-based interventions should not be interpreted as weak efficacy; rather, they likely reflect a higher proportion of patients with more severe asthma, who typically show less pronounced improvements than those with milder disease (30, 31). Notably, a large-scale randomized trial of an app-based intervention has reported improved symptom control over 12 months (8).

Sensitivity analyses suggested that the observed heterogeneity was primarily driven by variations in study populations and intervention intensity. Regarding the mini-AQLQ scores at 6 months, the study by Farzandipour et al. (20) emerged as a significant positive outlier, exhibiting a markedly larger effect size. This finding may be attributed to the RespRight application utilized in their study, which integrates a direct communication channel between patients and physicians, moving beyond a standard self-monitoring tool. This feature likely reinforced the clinical feedback loop and enhanced patient treatment adherence. Conversely, for both the mini-AQLQ and ACQ scores at 12 months, the study by Beerthuizen et al. (21) was identified as the primary source of heterogeneity and was the only study to report a lack of clinical benefit. This discrepancy may stem from their recruitment of severe asthma patients who had recently completed a 12-week high-altitude pulmonary rehabilitation program. The challenges of transitioning from a specialized clinical climate to a home environment, combined with the absence of direct clinician-led communication in the intervention, may have limited the efficacy of the digital tool within this specific high-risk population.

Influence of Intervention Components Beyond delivery modality, the observed efficacy was largely driven by the specific components of the digital interventions, including clinician intensity, hardware integration, and educational design First, clinician involvement appeared to be the strongest predictor of success. Interventions featuring a “closed-loop” feedback system with healthcare professionals consistently outperformed passive (20, 23), “open-loop” self-monitoring (21). The active involvement of clinicians allows for timely triage and medication adjustments, transforming collected data into actionable clinical decision; Second, regarding digital inhalers, Susanne et al. (10) demonstrated that hardware integration enhances data reliability. By providing objective medication-use records rather than relying on patient recall, digital inhalers facilitate evidence-based discussions on adherence, which is often the prerequisite for improved clinical outcomes; Third, educational components and program intensity also played a role. Unlike static brochures used in usual care, digital interventions delivered education in a more dynamic, high-intensity format (18, 26). This educational approach ensures that patients receive guidance precisely when their asthma control deteriorates, thereby improving self-management competence more effectively than standard education alone.

While the observed benefits are promising, translating short-term efficacy into long-term practice requires addressing the “law of attrition” observed in our review (intervention attrition: 15.65% vs. control: 9.06%). To sustain adherence, future interventions must incorporate robust engagement strategies while minimizing alert fatigue (32). In asthma, a systematic review of digital inhalers reported a median trial attrition of approximately 10.2% (33). Evidence from mHealth further indicates that adherence is influenced by intervention complexity, contextual relevance, personalization, and duration (34). Therefore, shifting toward passive data collection (e.g., via digital inhalers or wearables) and employing “just-in-time” adaptive notifications can reduce friction and maintain user interest over time.

Compared with previous evidence, our findings are consistent with the advantages of interactive, clinician-supported remote digital interventions. In COPD, multiple studies have shown that remote monitoring and follow-up can reduce readmissions and improve quality of life; however, overall effects are heterogeneous and appear to depend on program intensity and follow-up frequency (11, 35); In asthma, by contrast, stand-alone self-management tools or video-based education without clinician engagement generally yield only modest and short-lived benefits (9). Established asthma evidence further indicates that supported self-management combined with regular professional review sustainably improves control and reduces exacerbations (36). From a digital health perspective, remote care and wearable technologies are increasingly integrated with deep learning-based AI for exacerbation risk prediction, personalized intervention prompts, and risk stratification, which may enhance both the effectiveness and adherence of remote management programs (7, 37). Finally, given the heterogeneity in patient needs and the costs associated with digital devices, patient stratification is essential for scalable implementation (38). Prior evidence indicates that digital inhalers can improve maintenance-medication adherence, reduce reliever use, and, in some cases, sustain improvements in asthma control over 12 months (33, 39). However, these resource-intensive tools should not be applied uniformly but rather stratified for high-risk or adherence-challenged populations (12). This targeted approach ensures that the intensity of digital monitoring matches the patient's clinical risk profile, optimizing both cost-effectiveness and clinical outcomes (40, 41).

Systemically, the shift to proactive home-based monitoring can alleviate healthcare burdens by reducing emergency visits. However, scalability relies on seamless interoperability with Electronic Health Records to avoid increasing clinician workload. To address equity, a stepped-care strategy is advisable: prioritizing accessible mobile apps for the general population while reserving resource-intensive technologies (e.g., digital inhalers) for high-risk patients, thereby balancing cost-effectiveness with clinical need.

## Limitations

This review has several limitations. First, the overall risk of bias among the included studies was high, particularly for patient-reported outcomes that could not be blinded, which may have led to overestimation of the effects on mini-AQLQ and ACQ. Second, the available evidence was limited at several endpoint time points, restricting the robustness of temporal inferences. Third, interventions were delivered via smartphone applications or web-based platforms with substantial variability in intervention intensity, parameters recorded and reported, and the extent of clinician involvement, thereby limiting comparability across studies. Fourth, frequent remote reporting tasks and follow-up requirements were associated with higher attrition in the intervention groups, potentially introducing attrition bias and reducing the external validity of long-term adherence estimates. Finally, most trials were single-center and predominantly enrolled female participants, which may constrain the generalizability of the findings.

These limitations highlight the need for future randomized controlled trials with larger sample sizes, more rigorous control of bias, and standardized, clearly defined intervention components. Standardized and extended follow-up periods are also required to better assess long-term effects on quality of life, clinical outcomes, and healthcare costs. In addition, stratification by disease severity and the targeted use of wearable technologies such as digital inhalers, especially in high-risk or adherence-challenged populations, may help optimize both effectiveness and adherence.

## Conclusion

This review found that interactive remote digital management between patients and healthcare professionals resulted in small-to-moderate improvements that were statistically significant but did not meet the MCID criteria for patient-reported outcomes. Therefore, while digital interventions with closed-loop feedback are superior to usual care, their clinical impact on symptoms may be modest, particularly for short-term outcomes. Taking into account the risk of bias and heterogeneity, the overall certainty of the evidence was low to moderate. Overall, digital interventions that integrate clinical follow-up and closed-loop feedback outperform education alone and usual care; however, larger, multicenter randomized controlled trials with standardized intervention components and longer follow-up are still required to clarify the independent and interactive effects of delivery modality, co-implementation of digital inhalers, and the intensity of healthcare professional involvement, thereby further confirming the magnitude and stability of the observed effects.

## Data Availability

The original contributions presented in the study are included in the article/Supplementary material, further inquiries can be directed to the corresponding authors.
