# Supplementary material for: Digital, remotely delivered management interventions for adult asthma: a systematic review and meta-analysis
Source: Front Public Health. 2026 Mar 31;14:1776893. doi: 10.3389/fpubh.2026.1776893 (PMC13076491; doi:10.3389/fpubh.2026.1776893)
Supplement: Supplementary file 1 [file Data_Sheet_1.docx]

**Digital, Remotely Delivered Management Interventions for Adult Asthma: A Systematic Review and Meta-analysis**

**Supplement Materials**

Table 1S. Search strategies

| **Pubmed** | **Search time: 2025/9/7** |
| --- | --- |
| ("asthma"[Mesh] OR asthma*[Title/Abstract] OR "bronchial asthma"[Title/Abstract] OR "atopic asthma"[Title/Abstract] OR "cough variant asthma"[Title/Abstract] OR "allergic asthma"[Title/Abstract] OR "acute asthma"[Title/Abstract] OR "persistent asthma"[Title/Abstract] OR asthmatic*[Title/Abstract] OR wheezing[Title/Abstract] OR cough[Title/Abstract])  ("telemedicine"[MeSH Terms] OR telemedicine[tiab] OR telehealth[tiab] OR "remote consultation"[tiab] OR "remote monitoring"[tiab] OR "electronic monitoring device"[tiab] OR "smart inhaler"[tiab] OR "electronic inhaler monitoring"[tiab] OR "inhaler tracking"[tiab] OR "digital intervention"[tiab] OR "digital health"[tiab] OR "digital support"[tiab] OR "digital coaching"[tiab] OR "self-management program"[tiab] OR "asthma self-management"[tiab] OR "behavioral intervention"[tiab] OR "mobile application"[tiab] OR mHealth[tiab] OR eHealth[tiab] OR "wearable"[tiab] OR "wearable device"[tiab] OR "home monitoring"[tiab] OR "home-based monitoring"[tiab] OR "environmental monitoring"[tiab] OR "air quality sensor"[tiab] OR "virtual clinic"[tiab] OR "video consultation"[tiab] OR "online education"[tiab] OR "tele-rehabilitation"[tiab])  AND  ("usual care" OR "standard care" OR "standard therapy" OR "routine care" OR "usual treatment"  OR "face-to-face care" OR "conventional management" OR "usual clinical management" OR "standard follow-up"  OR "control group" OR "placebo" OR "sham intervention" OR "minimal intervention" OR "education only")  AND  ("Asthma Control Test" OR ACT OR "Asthma Control Questionnaire" OR ACQ OR "asthma control" OR "disease control"  OR exacerbation OR "asthma exacerbation" OR "severe exacerbation" OR hospitalization OR "emergency visit" OR "emergency department"  OR "medication adherence" OR adherence OR compliance OR "inhaler adherence"  OR "Asthma Quality of Life Questionnaire" OR AQLQ OR "Mini-AQLQ" OR "quality of life" OR "health-related quality of life" OR HRQoL  OR FEV1 OR "forced expiratory volume" OR "peak expiratory flow" OR PEF OR "lung function"  OR "healthcare utilization" OR "hospital admission" OR "medical resource use" OR "cost-effectiveness" OR "healthcare cost")  AND  (randomized controlled trial[pt] OR controlled clinical trial[pt] OR randomized[tiab] OR placebo[tiab] OR "drug therapy"[sh] OR randomly[tiab] OR trial[tiab] OR groups[tiab]) | |
| **Cochrane** | **Search time: 2025/9/7** |
| ("asthma"[Mesh] OR asthma*[Title/Abstract] OR "bronchial asthma"[Title/Abstract] OR "atopic asthma"[Title/Abstract] OR "cough variant asthma"[Title/Abstract] OR "allergic asthma"[Title/Abstract] OR "acute asthma"[Title/Abstract] OR "persistent asthma"[Title/Abstract] OR asthmatic*[Title/Abstract] OR wheezing[Title/Abstract] OR cough[Title/Abstract])  ("telemedicine"[MeSH Terms] OR telemedicine[tiab] OR telehealth[tiab] OR "remote consultation"[tiab] OR "remote monitoring"[tiab] OR "electronic monitoring device"[tiab] OR "smart inhaler"[tiab] OR "electronic inhaler monitoring"[tiab] OR "inhaler tracking"[tiab] OR "digital intervention"[tiab] OR "digital health"[tiab] OR "digital support"[tiab] OR "digital coaching"[tiab] OR "self-management program"[tiab] OR "asthma self-management"[tiab] OR "behavioral intervention"[tiab] OR "mobile application"[tiab] OR mHealth[tiab] OR eHealth[tiab] OR "wearable"[tiab] OR "wearable device"[tiab] OR "home monitoring"[tiab] OR "home-based monitoring"[tiab] OR "environmental monitoring"[tiab] OR "air quality sensor"[tiab] OR "virtual clinic"[tiab] OR "video consultation"[tiab] OR "online education"[tiab] OR "tele-rehabilitation"[tiab])  AND  ("usual care" OR "standard care" OR "standard therapy" OR "routine care" OR "usual treatment"  OR "face-to-face care" OR "conventional management" OR "usual clinical management" OR "standard follow-up"  OR "control group" OR "placebo" OR "sham intervention" OR "minimal intervention" OR "education only")  AND  ("Asthma Control Test" OR ACT OR "Asthma Control Questionnaire" OR ACQ OR "asthma control" OR "disease control"  OR exacerbation OR "asthma exacerbation" OR "severe exacerbation" OR hospitalization OR "emergency visit" OR "emergency department"  OR "medication adherence" OR adherence OR compliance OR "inhaler adherence"  OR "Asthma Quality of Life Questionnaire" OR AQLQ OR "Mini-AQLQ" OR "quality of life" OR "health-related quality of life" OR HRQoL  OR FEV1 OR "forced expiratory volume" OR "peak expiratory flow" OR PEF OR "lung function"  OR "healthcare utilization" OR "hospital admission" OR "medical resource use" OR "cost-effectiveness" OR "healthcare cost")  AND  (randomized controlled trial[pt] OR controlled clinical trial[pt] OR randomized[tiab] OR placebo[tiab] OR "drug therapy"[sh] OR randomly[tiab] OR trial[tiab] OR groups[tiab]) | |
| **Web of Science** | **Search time: 2025/9/7** |
| TS=("asthma" OR asthma* OR "bronchial asthma" OR "atopic asthma" OR "cough variant asthma" OR "allergic asthma" OR "acute asthma" OR "persistent asthma" OR asthmatic* OR wheezing OR cough) TS=("telemedicine" OR telemedicine OR telehealth OR "remote consultation" OR "remote monitoring" OR "electronic monitoring device" OR "smart inhaler" OR "electronic inhaler monitoring" OR "inhaler tracking" OR "digital intervention" OR "digital health" OR "digital support" OR "digital coaching" OR "self-management program" OR "asthma self-management" OR "behavioral intervention" OR "mobile application" OR mHealth OR eHealth OR "wearable" OR "wearable device" OR "home monitoring" OR "home-based monitoring" OR "environmental monitoring" OR "air quality sensor" OR "virtual clinic" OR "video consultation" OR "online education" OR "tele-rehabilitation")  AND  ALL= ("usual care" OR "standard care" OR "standard therapy" OR "routine care" OR "usual treatment"  OR "face-to-face care" OR "conventional management" OR "usual clinical management" OR "standard follow-up"  OR "control group" OR "placebo" OR "sham intervention" OR "minimal intervention" OR "education only")  AND  ALL=("Asthma Control Test" OR ACT OR "Asthma Control Questionnaire" OR ACQ OR "asthma control" OR "disease control"  OR exacerbation OR "asthma exacerbation" OR "severe exacerbation" OR hospitalization OR "emergency visit" OR "emergency department"  OR "medication adherence" OR adherence OR compliance OR "inhaler adherence"  OR "Asthma Quality of Life Questionnaire" OR AQLQ OR "Mini-AQLQ" OR "quality of life" OR "health-related quality of life" OR HRQoL  OR FEV1 OR "forced expiratory volume" OR "peak expiratory flow" OR PEF OR "lung function"  OR "healthcare utilization" OR "hospital admission" OR "medical resource use" OR "cost-effectiveness" OR "healthcare cost")  AND  TS=("randomized controlled trial" OR "controlled clinical trial" OR randomized OR placebo OR "drug therapy" OR randomly OR trial OR groups) | |
| **Scopus** | **Search time: 2025/9/7** |
| ( TITLE-ABS-KEY ( ( "randomized controlled trial" OR "controlled clinical trial" OR randomized OR placebo OR "drug therapy" OR randomly OR trial OR groups ) ) ) AND ( ALL ( ( "Asthma Control Test" OR ACT OR "Asthma Control Questionnaire" OR ACQ OR "asthma control" OR "disease control" OR exacerbation OR "asthma exacerbation" OR "severe exacerbation" OR hospitalization OR "emergency visit" OR "emergency department" OR "medication adherence" OR adherence OR compliance OR "inhaler adherence" OR "Asthma Quality of Life Questionnaire" OR AQLQ OR "Mini-AQLQ" OR "quality of life" OR "health-related quality of life" OR HRQoL OR FEV1 OR "forced expiratory volume" OR "peak expiratory flow" OR PEF OR "lung function" OR "healthcare utilization" OR "hospital admission" OR "medical resource use" OR "cost-effectiveness" OR "healthcare cost" ) ) ) AND ( ALL ( ( "usual care" OR "standard care" OR "standard therapy" OR "routine care" OR "usual treatment" OR "face-to-face care" OR "conventional management" OR "usual clinical management" OR "standard follow-up" OR "control group" OR "placebo" OR "sham intervention" OR "minimal intervention" OR "education only" ) ) ) AND ( TITLE-ABS-KEY ( ( "telemedicine" OR telemedicine OR telehealth OR "remote consultation" OR "remote monitoring" OR "electronic monitoring device" OR "smart inhaler" OR "electronic inhaler monitoring" OR "inhaler tracking" OR "digital intervention" OR "digital health" OR "digital support" OR "digital coaching" OR "self-management program" OR "asthma self-management" OR "behavioral intervention" OR "mobile application" OR mHealth OR eHealth OR "wearable" OR "wearable device" OR "home monitoring" OR "home-based monitoring" OR "environmental monitoring" OR "air quality sensor" OR "virtual clinic" OR "video consultation" OR "online education" OR "tele-rehabilitation" ) ) ) AND ( TITLE-ABS-KEY ( ( "asthma" OR asthma* OR "bronchial asthma" OR "atopic asthma" OR "cough variant asthma" OR "allergic asthma" OR "acute asthma" OR "persistent asthma" OR asthmatic* OR wheezing OR cough ) ) ) | |
| **Embase** | **Search time: 2025/9/7** |
| ('asthma'/exp OR asthma*:ti,ab OR 'bronchial asthma':ti,ab OR 'atopic asthma':ti,ab OR 'cough variant asthma':ti,ab OR 'allergic asthma':ti,ab OR 'acute asthma':ti,ab OR 'persistent asthma':ti,ab OR asthmatic*:ti,ab OR wheezing:ti,ab OR cough:ti,ab)  ('telemedicine'/exp OR telemedicine:ti,ab OR telehealth:ti,ab OR 'remote consultation':ti,ab OR 'remote monitoring':ti,ab OR 'electronic monitoring device':ti,ab OR 'smart inhaler':ti,ab OR 'electronic inhaler monitoring':ti,ab OR 'inhaler tracking':ti,ab OR 'digital intervention':ti,ab OR 'digital health':ti,ab OR 'digital support':ti,ab OR 'digital coaching':ti,ab OR 'self-management program':ti,ab OR 'asthma self-management':ti,ab OR 'behavioral intervention':ti,ab OR 'mobile application':ti,ab OR mHealth:ti,ab OR eHealth:ti,ab OR 'wearable':ti,ab OR 'wearable device':ti,ab OR 'home monitoring':ti,ab OR 'home-based monitoring':ti,ab OR 'environmental monitoring':ti,ab OR 'air quality sensor':ti,ab OR 'virtual clinic':ti,ab OR 'video consultation':ti,ab OR 'online education':ti,ab OR 'tele-rehabilitation':ti,ab)  AND  ("usual care" OR "standard care" OR "standard therapy" OR "routine care" OR "usual treatment"  OR "face-to-face care" OR "conventional management" OR "usual clinical management" OR "standard follow-up" OR "control group" OR placebo OR "sham intervention" OR "minimal intervention" OR "education only")  AND  ('Asthma Control Test'/exp OR ACT OR 'Asthma Control Questionnaire' OR ACQ OR 'asthma control' OR 'disease control' OR exacerbation OR 'asthma exacerbation' OR 'severe exacerbation' OR hospitalization OR 'emergency visit' OR 'emergency department' OR 'medication adherence' OR adherence OR compliance OR 'inhaler adherence' OR 'Asthma Quality of Life Questionnaire' OR AQLQ OR 'Mini-AQLQ' OR 'quality of life' OR 'health-related quality of life' OR HRQoL OR FEV1 OR 'forced expiratory volume' OR 'peak expiratory flow' OR PEF OR 'lung function' OR 'healthcare utilization' OR 'hospital admission' OR 'medical resource use' OR 'cost-effectiveness' OR 'healthcare cost')  AND  ('randomized controlled trial'/exp OR 'controlled clinical trial'/exp OR randomized:ti,ab OR placebo:ti,ab OR 'drug therapy'/exp OR randomly:ti,ab OR trial:ti,ab OR groups:ti,ab) | |

Table 2S. Excluded studies from the original search with reasons for exclusion

| **First Author’s**  **Last Name** | **Title** | **Year** | **Reason for Exclusion** |
| --- | --- | --- | --- |
| Sakleshpur | Implementing and Evaluating the Clinical Outcomes of an Asthma Management Mobile Application | 2025 | Wrong Interventions  Wrong Outcomes |
| Eck | Effectiveness of an online asthma education program in adults with asthma in general practice: Results from a cluster randomised controlled trial | 2025 | Wrong Interventions |
| Chung | Smartphone App-Guided Pulmonary Rehabilitation in Chronic Respiratory Diseases: Randomized Controlled Trial | 2025 | Wrong Outcomes |
| Silberman | A Digital Asthma Self-Management Program for Adults: A Randomized Clinical Trial | 2025 | Wrong Outcomes |
| Thomas | Evaluating the ASTHMAXcel Mobile Application's Implementation Within Primary Care Settings Through the RE-AIM Framework | 2024 | Conference |
| Şimşek | The effectiveness of telemedicine methods in the follow-up of asthma in the COVID-19 pandemic process | 2024 | Wrong Interventions |
| Kentis | Assessing the Patient-Facing ASTHMAXcel Mobile Application Through the Health IT Evaluation Framework | 2024 | Conference |
| Kandola | Digital Self-Management Platform for Adult Asthma: Randomized Attention-Placebo Controlled Trial | 2024 | Wrong Interventions  Wrong Follow-up Period |
| Ghozali | Impact of a mobile-app assisted self-management educational intervention on the scores of asthma control test (ACT) questionnaire among young asthmatic patients | 2023 | Wrong Interventions  Wrong Follow-up Period |
| Mammen | Going mobile with primary care: smartphone-telemedicine for asthma management in young urban adults (TEAMS) | 2022 | Wrong Interventions |
| Agrawal | To assess asthma control using act and PEFR as home monitoring tool | 2022 | Conference |
| Onnis | Effect of pharmacy-supported digital medicine program on asthma control | 2022 | Conference |
| Iamlaor | Effectiveness of asthma self-care program through mobile Line application (SALA) on lung function among asthma patients in Angthong Hospital: A randomized control trial | 2021 | Wrong Interventions |
| Khusial | Effectiveness of myAirCoach: A mHealth Self-Management System in Asthma | 2020 | Wrong Interventions |
| McGihon | Effects of an asthma mHealth system on health services use: A pragmatic trial | 2019 | Conference |
| Ljungberg | Clinical effect on uncontrolled asthma using a novel digital automated self-management solution: A physician-blinded randomised controlled crossover trial | 2019 | Wrong Interventions  Wrong Follow-up Period |
| Khusial | Myaircoach: mHealth assisted self-management in patients with uncontrolled asthma, a randomized control trial | 2019 | Conference |
| Federman | Effect of a Self-management Support Intervention on Asthma Outcomes in Older Adults: The SAMBA Study Randomized Clinical Trial | 2019 | Wrong Interventions |
| Ainsworth | Investigating the feasibility of a mobile mindfulnessbased digital intervention for patients with asthma | 2018 | Conference |
| Weinstein | Preliminary evaluation of an adult asthma adherence management program | 2017 | Conference |
| Reece | Pilot study of asthmawin mobile iphone app in the management of asthma | 2017 | Conference |
| Zairina | Telehealth to improve asthma control in pregnancy: A randomized controlled trial | 2016 | Inappropriate Research Subjects |
| Rashid | Effectiveness of pictorial based self-management among adult with asthma in a suburban primary care health clinic: A randomised controlled trial | 2016 | Wrong Interventions  Wrong Follow-up Period |
| Olivera | Asthma self-management model: randomized controlled trial | 2016 | Wrong Follow-up Period |
| Merchant | Effectiveness of Population Health Management Using the Propeller Health Asthma Platform: A Randomized Clinical Trial | 2016 | Wrong Interventions  Inappropriate Research Subjects |
| Merchant | Interim results from a randomized, controlled trial of remote monitoring of inhaled bronchodilator use on asthma control, symptoms and management | 2014 | Conference |
| Araújo | Clinical efficacy of web-based versus standard Asthma self-management | 2012 | Wrong Control Group |
| Ryan | Clinical and cost effectiveness of mobile phone supported self monitoring of asthma: Multicentre randomised controlled trial | 2012 | Wrong Interventions  Inappropriate Research Subjects |

Table 3S. Baseline characteristics of the included trials

| Study ID | Estimated Sample Size  (intervention /control) | Completed Sample Size  (intervention /control) | Mean ages  (intervention /control) | No of male individuals  (intervention /control) |
| --- | --- | --- | --- | --- |
| Xu 2025(1) | 60/60 | 53/50 | 42.89/44.3 | 24/25 |
| Susanne 2025(2) | 82/82 | 68/77 | 47.5/47 | 68/36 |
| Robert 2025(3) | 211/202 | 176/190 | 51.9/52.6 | 54/38 |
| Mehrdad 2024(4) | 30/30 | 30/30 | 39.03/40.4 | 11/10 |
| Beerthuizen 2020(5) | 45/47 | 33/29 | 46.7/44 | 8/9 |
| Ben 2019(6) | 44/44 | 29/43 | 57/56.3 | 17/18 |
| Tiva 2019(7) | 51/49 | 49/49 | 48.33/51 | 24/24 |
| Sara 2016(8) | 49/51 | 32/48 | / | 15/18 |
| Johanna 2013(9) | 101/99 | 91/85 | 36/37 | / |
| Victor 2009(10) | 101/99 | 91/92 | 36/37 | 32/29 |

Table 4S. Summary of findings according to the GRADE approach.

| **Certainty assessment** | | | | | | | **Number of patients** | | **Absolute (95% CI)** | **Certainty** |
| --- | --- | --- | --- | --- | --- | --- | --- | --- | --- | --- |
| **Number of studies** | **Study design** | **Risk of bias** | **Inconsistency** | **Indirectness** | **Imprecision** | **Other considerations** | **Smartphone/website-based remote management** | **usual care** |  |  |
| **ACQ 12 months** | | | | | | | | | | |
| 5 | randomised trials | serious^a^ | serious^b^ | not serious | serious^c^ | none | 252 | 280 | MD 0.37 SD lower (0.67 lower to 0.13 lower) | ⨁◯◯◯ Very low^a,b,c^ |
| **FEV1% 12 months** | | | | | | | | | | |
| 5 | randomised trials | not serious | not serious | not serious | not serious | none | 235 | 252 | MD 4.49 SD higher (1.11 higher to 7.87 higher) | ⨁⨁⨁⨁ High |
| **Mini-AQLQ 6 months** | | | | | | | | | | |
| 5 | randomised trials | serious^d^ | serious^e^ | not serious | serious^f^ | none | 202 | 232 | MD 0.47 SD higher (0.02 higher to 0.92 higher) | ⨁◯◯◯ Very low^d,e,f^ |
| **Mini-AQLQ 12 months** | | | | | | | | | | |
| 6 | randomised trials | serious^d,g^ | not serious | not serious | not serious | none | 424 | 471 | MD 0.35 SD higher (0.21 higher to 0.49 higher) | ⨁⨁⨁◯ Moderate^d,g^ |

a. The outcome was the patient-reported ACQ, with a high risk of measurement bias.

b. Heterogeneity was high (I² = 73.3%, p = 0.005), indicating marked between-study differences in effect sizes.

c. The 95% CI excludes no effect but spans trivial to clinically important improvement versus the ACQ MCID, leaving uncertainty in the true effect size.

d. For mini-AQLQ, concerns mainly arise from the measurement domain (unblinded patient-reported outcome) and from deviations from intended interventions/inappropriate analysis.

e. the heterogeneity was substantial (I² = 70.6%, p = 0.01) and could not be adequately explained.

f. The 95% CI (0.02–0.92) excludes no effect but ranges from a trivial to a clinically important benefit, indicating uncertainty in the effect size.

CI: confidence interval; SMD: standardised mean difference

Figure 1S. Risk of Bias 2 assessment for each item and included study.

Figure 2S. Summary of risk of bias 2 assessment.


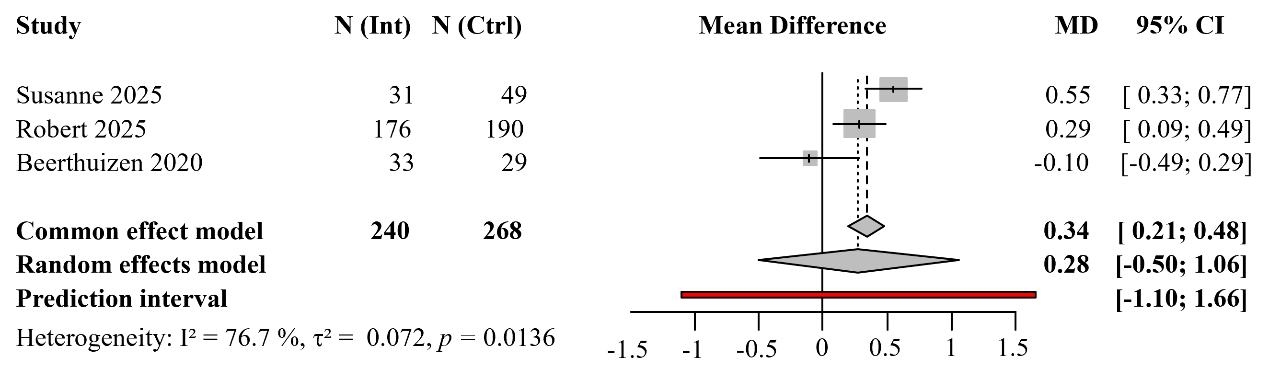


Figure 3S. Random Effects Model Analysis of Mini-AQLQ at 12 Months Based on Smartphone App Intervention.


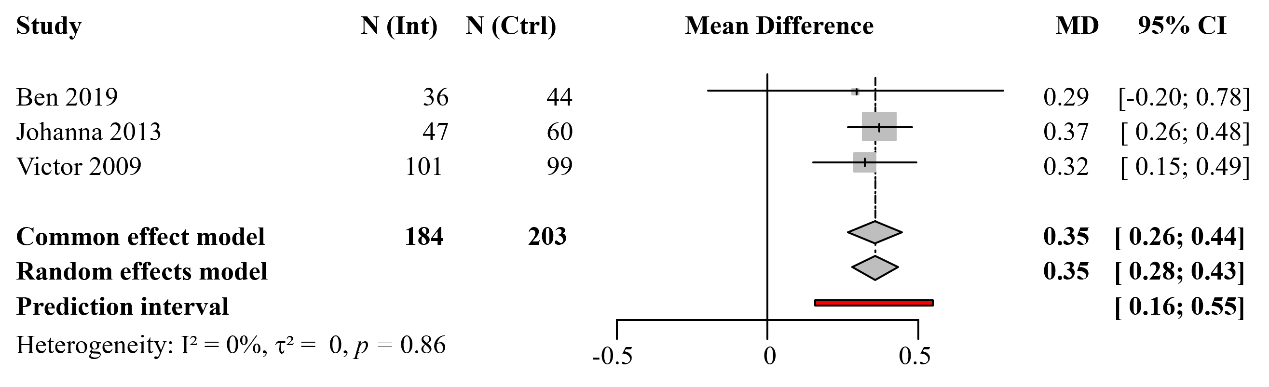


Figure 4S. Random Effects Model Analysis of Mini-AQLQ at 12 Months Based on Web-Based Intervention.


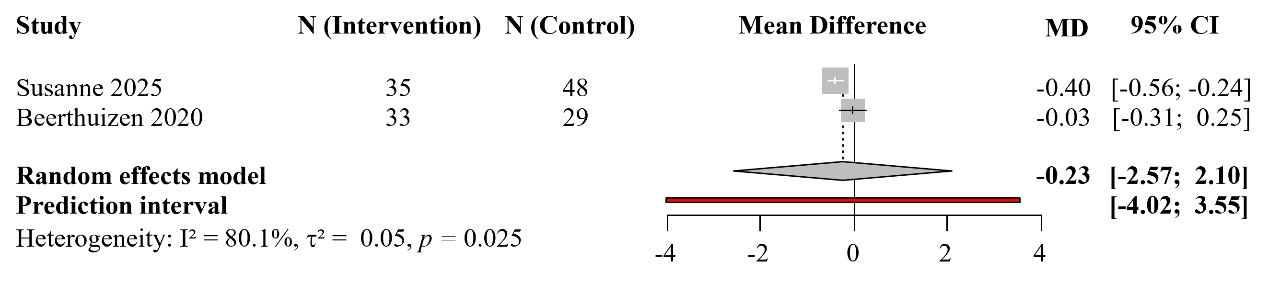


Figure 5S. Random Effects Model Analysis of ACQ at 12 Months Based on Smartphone App Intervention.


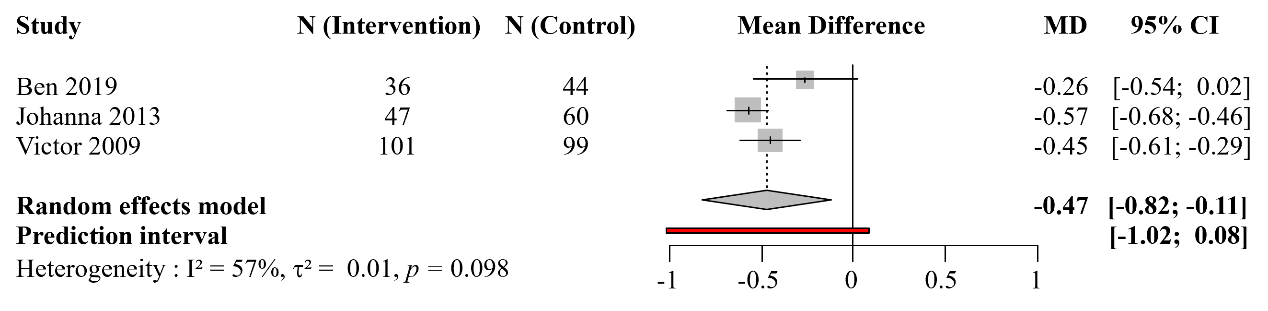


Figure 6S. Random Effects Model Analysis of ACQ at 12 Months Based on Web-Based Intervention.


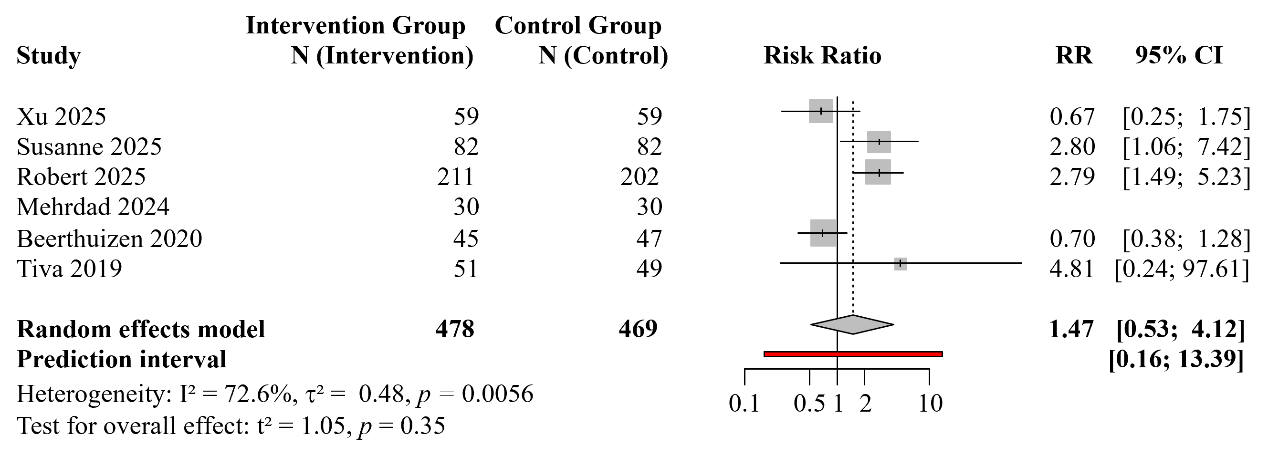


Figure 7S. Loss to follow-up rate in the smartphone app group


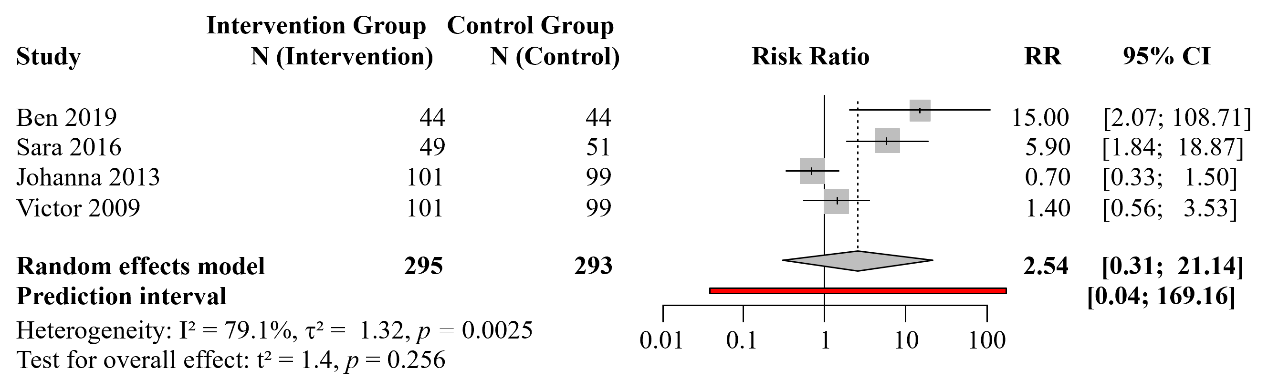


Figure 8S. Loss to follow-up rate in the web-based group


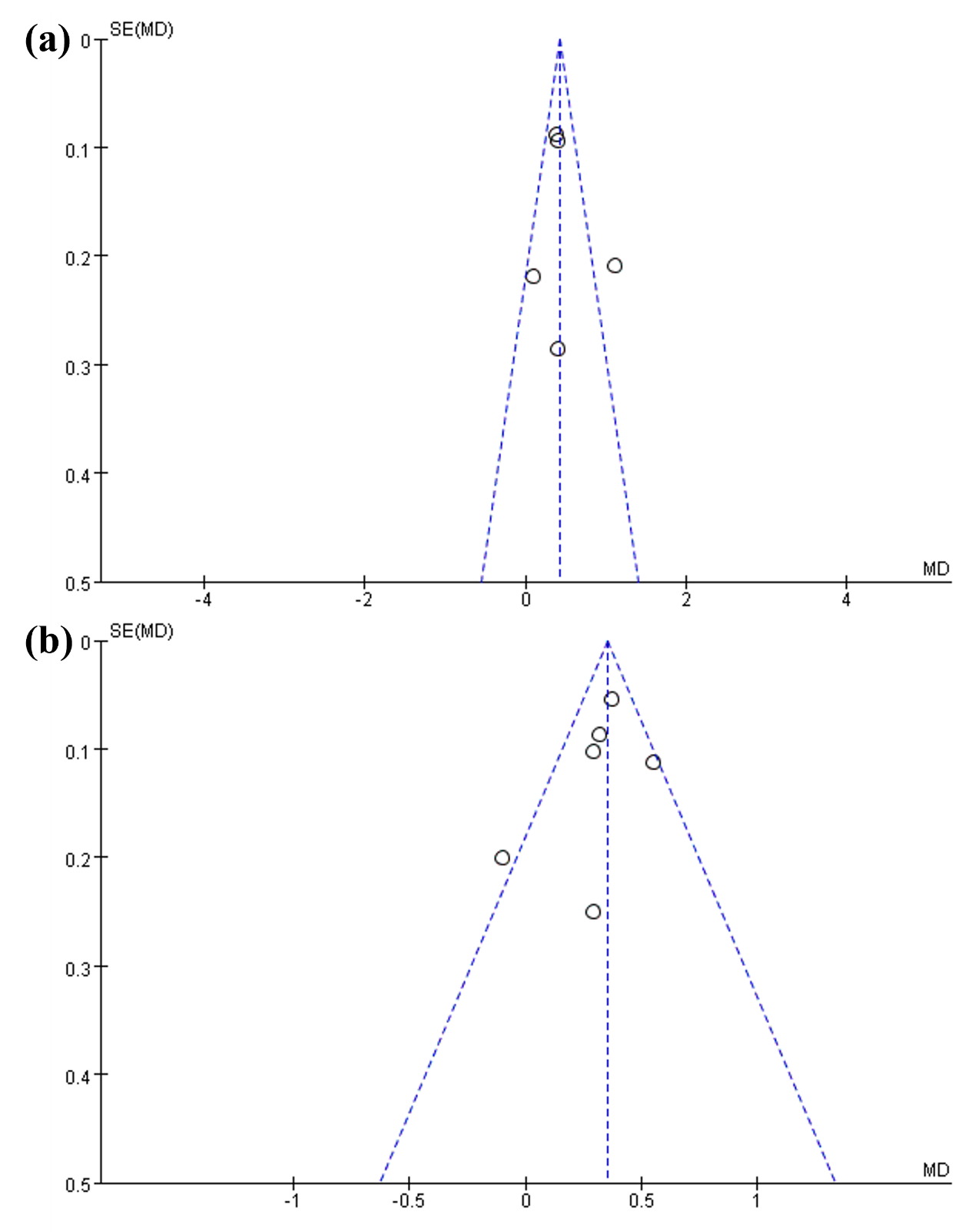


Figure 9S. Funnel plots for small-study effects in AQLQ. (a) AQLQ at 6 months; (b) AQLQ at 12 months.


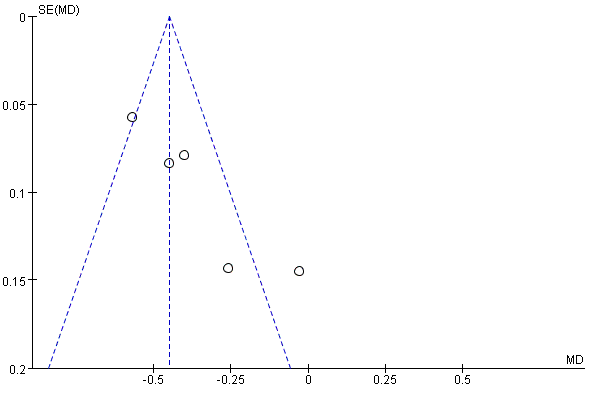


Figure 10S. Funnel plot for small-study effects in ACQ at 12 months.


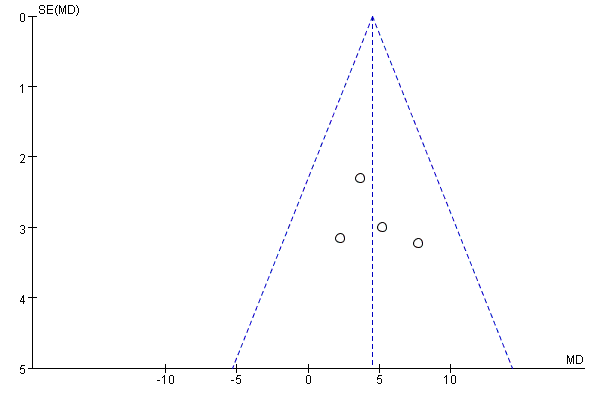


Figure 11S. Funnel plot for small-study effects in FEV_1_% at 12 months.


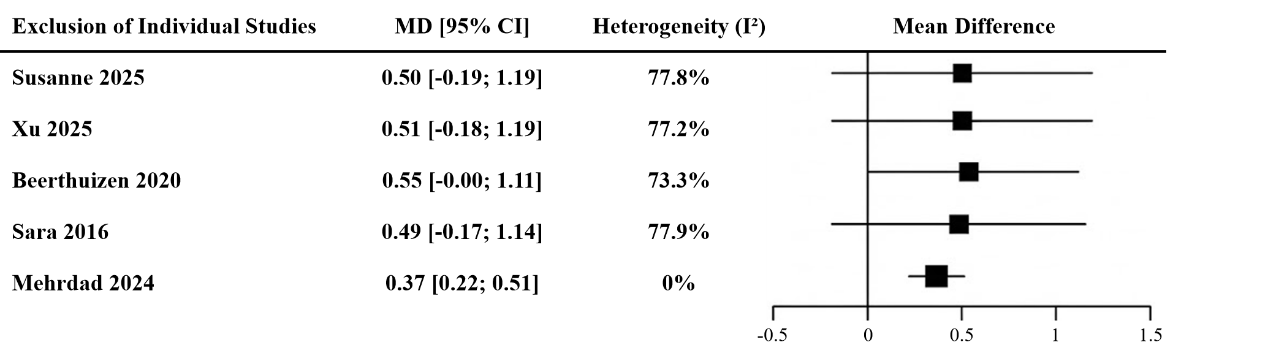


Figure 12S. Leave-one-out sensitivity analysis for Mini-AQLQ scores at 6 months.


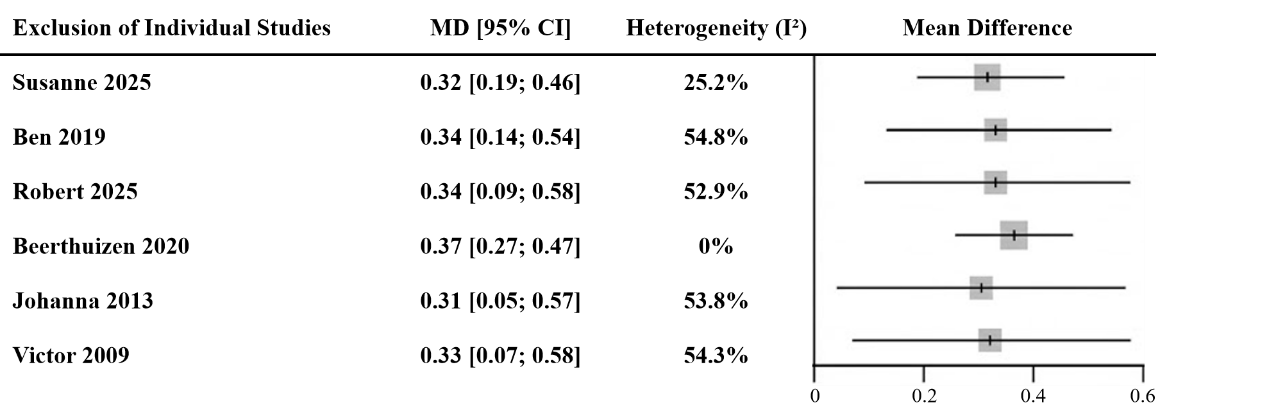


Figure 13S. Leave-one-out sensitivity analysis for Mini-AQLQ scores at 12 months.


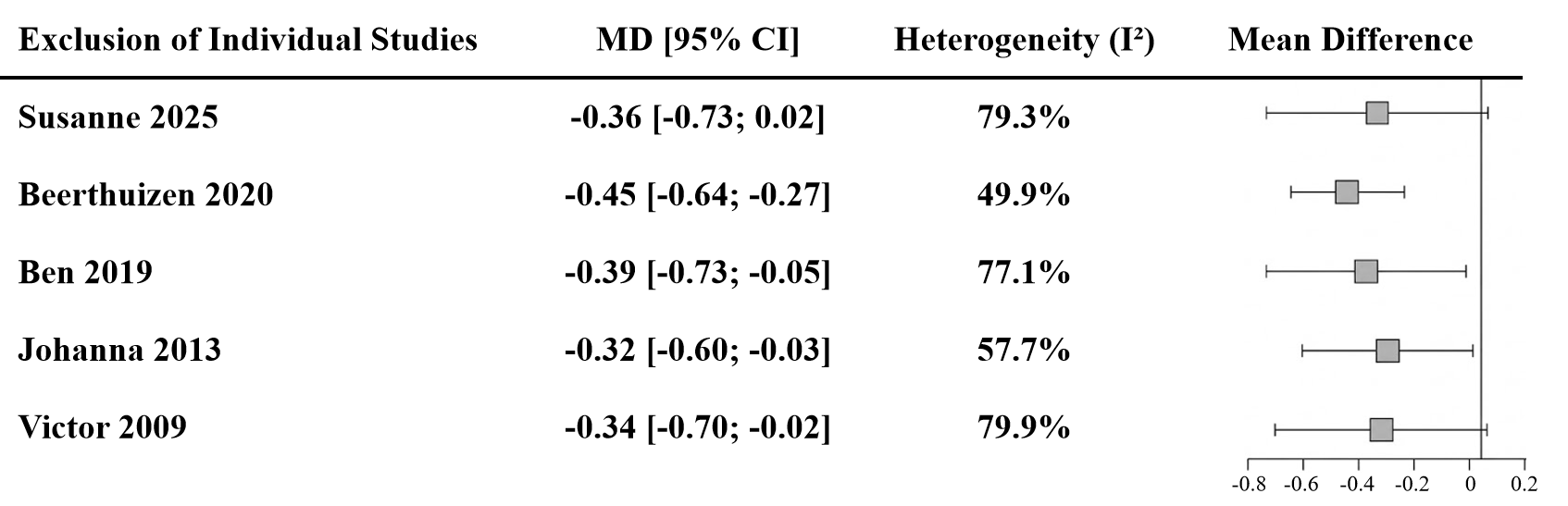


Figure 14S. Leave-one-out sensitivity analysis for ACQ scores at 12 months.

**References**

1. Xu S, Song Z, Cheng X, Wang J. An asthma self-management program based on WeChat to improve asthma control and quality of life: a randomized controlled trial. Frontiers in Allergy. 2025;6.

2. van de Hei SJ, van den Berg LN, Poot CC, Gerritsma YH, Meijer E, Flokstra-de Blok BMJ, et al. Long-Term Effectiveness of a Digital Inhaler on Medication Adherence and Clinical Outcomes in Adult Asthma Patients in Primary Care: the Cluster Randomized Controlled ACCEPTANCE Trial. The journal of allergy and clinical immunology In practice. 2025;13(7):1693‐704.

3. Rudin RS, Plombon S, Sulca Flores J, Sousa JL, Rodriguez J, Foer D, et al. Between-Visit Asthma Symptom Monitoring With a Scalable Digital Intervention: A Randomized Clinical Trial. JAMA Network Open. 2025;8(4).

4. Farzandipour M, Heidarzadeh M, Sharif R, Nabovati E, Akbari H, Anvari S. Improving asthma control and quality of life via a smartphone self-management app: A randomized controlled trial. Respiratory Medicine. 2024;223.

5. Beerthuizen T, Rijssenbeek-Nouwens LH, van Koppen SM, Khusial RJ, Snoeck-Stroband JB, Sont JK. Internet-Based Self-Management Support After High-Altitude Climate Treatment for Severe Asthma: Randomized Controlled Trial. J Med Internet Res. 2020;22(7):e13145.

6. Ainsworth B, Greenwell K, Stuart B, Raftery J, Mair F, Bruton A, et al. Feasibility trial of a digital self-management intervention 'My Breathing Matters' to improve asthma-related quality of life for UK primary care patients with asthma. Bmj Open. 2019;9(11).

7. Nemanič T, Sarc I, Skrgat S, Fležar M, Cukjati I, Marc-Malovrh M. Telemonitoring in asthma control: a randomized controlled trial. Journal of Asthma. 2019;56(7):782-90.

8. Ahmed S, Ernst P, Bartlett SJ, Valois MF, Zaihra T, Paré G, et al. The Effectiveness of Web-Based Asthma Self-Management System, My Asthma Portal (MAP): A Pilot Randomized Controlled Trial. J Med Internet Res. 2016;18(12):e313.

9. van Gaalen JL, Beerthuizen T, van der Meer V, van Reisen P, Redelijkheid GW, Snoeck-Stroband JB, et al. Long-Term Outcomes of Internet-Based Self-Management Support in Adults With Asthma: Randomized Controlled Trial. Journal of Medical Internet Research. 2013;15(9):40-52.

10. Van Der Meer V, Bakker MJ, Van Den Hout WB, Rabe KF, Sterk PJ, Kievit J, et al. Internet-based self-management plus education compared with usual care in asthma: A randomized trial. Annals of Internal Medicine. 2009;151(2):110-20.
